# Fabrication and Characterization of Magnesium Ferrite-Based PCL/Aloe Vera Nanofibers

**DOI:** 10.3390/ma10080937

**Published:** 2017-08-11

**Authors:** Zanshe Thompson, Shekh Rahman, Sergey Yarmolenko, Jagannathan Sankar, Dhananjay Kumar, Narayan Bhattarai

**Affiliations:** 1 Department of Chemical, Biological and Bioengineering, North Carolina A&T State University, Greensboro, NC 27411, USA; zsthomps@aggies.ncat.edu (Z.T.); srahman@aggies.ncat.edu (S.R.); 2 NSF Engineering Research Center for Revolutionizing Metallic Biomaterials, Greensboro, NC 27411, USA; sergey@ncat.edu (S.Y.); sankar@ncat.edu (J.S.); dkumar@ncat.edu (D.K.); 3 Department of Mechanical Engineering, North Carolina A&T State University, Greensboro, NC 27411, USA

**Keywords:** magnesium ferrite, electrospinning, scaffolds, magnetic nanofibers, PCL, aloe vera

## Abstract

Composite nanofibers of biopolymers and inorganic materials have been widely explored as tissue engineering scaffolds because of their superior structural, mechanical and biological properties. In this study, magnesium ferrite (Mg-ferrite) based composite nanofibers were synthesized using an electrospinning technique. Mg-ferrite nanoparticles were first synthesized using the reverse micelle method, and then blended in a mixture of polycaprolactone (PCL), a synthetic polymer, and Aloe vera, a natural polymer, to create magnetic nanofibers by electrospinning. The morphology, structural and magnetic properties, and cellular compatibility of the magnetic nanofibers were analyzed. Mg-ferrite/PCL/Aloe vera nanofibers showed good uniformity in fiber morphology, retained their structural integrity, and displayed magnetic strength. Experimental results, using cell viability assay and scanning electron microscopy imaging showed that magnetic nanofibers supported 3T3 cell viability. We believe that the new composite nanofibrous membranes developed in this study have the ability to mimic the physical structure and function of tissue extracellular matrix, as well as provide the magnetic and soluble metal ion attributes in the scaffolds with enhanced cell attachment, and thus improve tissue regeneration.

## 1. Introduction

Electrospinning technique is an increasingly popular option for creating engineered composite nanofibers out of variety of different polymers and metal–ceramics particles for tissue engineering scaffold design. Among the various techniques for nanofiber fabrication, which include melt blowing, phase separation, self-assembly, and template synthesis, electrospinning is relatively simple, inexpensive and reliable [1,2]. Electrospinning is a versatile technique that enables the engineering of scaffolds with micro-to-nanoscale topography and with porosity that can be tuned to match the tissue extracellular matrix. Nanofibers in a scaffold offer guidance cues that result in cell outgrowth, such as neurite and muscle bundles, in the direction of the nanofibers [3,4]. This is possibly due to favorable interactions between cell filopodia and nanofibers, which are similar in diameter [5]. The proper selection of scaffold materials is a key factor to determine the efficacy of nanofibers in specific tissue engineering applications. The material degradation rate, mechanical properties, and ability to guide cells to regenerate tissues are important properties for the polymers chosen [6,7]. 

Magnetic nanoparticles have many biomedical applications, including cell tracking, protein and DNA separation, targeted drug delivery, hyperthermic treatment, and tissue regeneration [8,9]. The main advantages of magnetic nanocrystals are low interference, low background signal, no necessity for pre-treatment, and portability, due to their size [10]. Magnetic nanocrystals have been reported to have property and size similarities to biological molecules [9]. Ferrite nanocrystals are of great interest due to their magnetic properties, relative non-toxicity, biodegradability, biocompatibility, and high surface-to-volume ratio [3,11,12]. Iron oxide nanoparticles have been reported to increase neural and mucosal cell growth after being incorporated into hydrogel scaffolds [13]. Magnesium ferrite (Mg-ferrite, MgFe_2_O_4_), a soft magnetic semiconducting material, has recently gained popularity in biomedical engineering, particularly in tissue engineering applications [14,15]. Mg-ferrite belongs to the same spinel ferrite group as Fe_3_O_2_, and has the added benefit of magnesium ions. Magnesium has been shown to catalyze enzymatic pathways, and promote cell growth and proliferation [16,17]. Iron oxide-based nanocrystals have been found to enhance the neurite outgrowth due to their magnetic and conductive properties [18,19]. The exposure of PC12, a neuron-like cell line, to both iron oxide nanoparticles and nerve growth factor has been shown to synergistically increase the efficacy of neurite outgrowth in a dose-dependent manner [18]. 

Magnetic nanofibers have recently sparked interest in the field of biomedical engineering due to their various potential applications, which include biosensing, targeted drug delivery, bone tissue regeneration, and DNA separation [20,21]. The incorporation of magnetic nanoparticles into nanofibers is expected to enhance their usability in tissue engineering due to the synergistic effect obtained from both the nanofibers and magnetic nanoparticles [20,22]. Polycaprolactone (PCL), a synthetic polymer, is well known in electrospinning for its mechanical strength, biodegradability, and miscibility with a variety of other polymers to produce stable polyblends [5,23,24]. However, PCL exhibits poor cellular response due to its lack of cell affinity [25]. Aloe vera, a natural polymer, has received considerable attention in biomedical engineering due to its numerous beneficial effects. Aloe vera powder, obtained from Aloe vera gel, contains over 75 biologically active and naturally-occurring compounds, including polysaccharides, amino acids, vitamins, lipids, sterols, and minerals [26,27]. The polysaccharides (e.g., acemannan and glucomannan) are responsible for the majority of the functional activities observed from the use of the Aloe vera plant [28]. However, the functional activities of Aloe vera result from the synergistic action of a variety of compounds that have been shown to promote cell migration, proliferation and growth [1,27]. Blends of synthetic and natural polymers can form a new class of biomaterials with improved mechanical properties and biocompatibility compared with those of single components [29]. 

In this work, Mg-ferrite based PCL/Aloe vera nanofibers were synthesized. Mg-ferrite nanoparticles were first synthesized using the reverse micelle method, and then blended in a mixture of PCL and Aloe vera solution. Electrospinning technique was utilized to transform the Mg-ferrite/PCL/Aloe vera blend solution into nanofibers. The potential use of these magnetic nanofibers was studied through analyzing several physicochemical properties such as their morphology, and structural and magnetic properties. The cellular compatibility of the magnetic nanofibers was studied by seeding 3T3 fibroblast cells on the fibers. Cell viability was quantified by alamarBlue assay, and cell attachment was characterized by scanning electron microscope (SEM). Mg-ferrite nanoparticles encapsulated in the PCL/Aloe vera nanofibers with paramagnetic properties could be potentially applied in several biomedical fields.

## 2. Materials and Methods

### 2.1. Preparation of Mg-Ferrite Nanoparticles

Mg-ferrite nanoparticles were prepared according to the previously reported reverse micelle method with some modifications [30]. Briefly, magnesium nitrate hexahydrate and iron (III) nitrate nonahydrate salts (Sigma Aldrich, St. Louis, MO, USA) were dissolved in deionized (DI) water at 1:2 molar ratio. 80 mL of poly(oxyethylene) nonylphenyl ether (Sigma Aldrich) and 200 mL of hexane were mixed with 39 mL of the aqueous salt solution. The solution was mixed rapidly for at least 5 min so that an equilibrium was achieved. Ammonium hydroxide (18 mL) was injected into the solution using two 10-mL pipettes. A deep brown precipitate was obtained. The precipitate was washed several times with 70% ethanol, and dried at 80 °C for 24 h in a drying oven. The dried product was crushed and calcined at 800 °C for 2 h.

### 2.2. Preparation of Mg-Ferrite Based PCL/Aloe Vera Nanofibers

PCL (Mn 80 kDa, Sigma Aldrich) solution was prepared by dissolving PCL pellets into 2,2,2-Trifluoroethanol (TFE, Alfa Aesar, Haverhill, MA, USA) at a concentration of 10 wt %. Aloe vera powder (Terry Laboratories, Melbourne, FL, USA, catalog number TN003) was dissolved in DI water at a concentration of 10 wt %. The Aloe vera solution was vortexed for 5 min to ensure that the Aloe vera was completely dissolved. Mg-ferrite nanoparticles were then dispersed in the PCL solution. When both of the solutions were dissolved, a plastic syringe was used to remove the appropriate amount of each solution to generate the 70:30 ratio of PCL/Aloe vera blend. The concentrations of Mg-ferrite nanoparticles in the PCL/Aloe vera solution were 5, 10, 15, 20 and 25 wt %. Prior to electrospinning, the solution mixture was sonicated to prepare a homogenous composite solution. Approximately 4 mL of polymer solution was loaded into a 5-mL disposable syringe fitted with a 1-mm diameter, stainless steel needle. The Spellman CZE1000R (Hauppauge, NY, USA) high-voltage power supply was clipped to the syringe needle tip. The high voltage power source was adjusted to a voltage of 7–18 kV, depending on the composition of the solution. The polymer solution was injected at a rate of 0.5 mL/h and electrospun at room temperature. The stationary syringe tip was approximately 10 cm away from a plastic, cylindrical grounded collector wrapped with aluminum foil. The motor, powered by a DC power source, rotated the collector at a constant speed.

### 2.3. Characterization of Nanoparticles and Nanofibers

The morphology of the nanoparticles was analyzed with a transmission electron microscope (TEM, Technai G2 Twin, Hillsboro, OR, USA) operating at an accelerated voltage of 200 kV. Powder X-ray diffraction (XRD) patterns were recorded on a D8 diffractometer (Bruker, Billerica, MA, USA). Raman spectra were measured at room temperature with an ARAMIS Raman Spectrometer (Horiba Scientific, Edison New Jersey, NJ, USA) using a 532 nm Ar-ion laser as the excitation source.

The morphology and diameter of the electrospun fibers were observed by scanning electron microscopy (SEM, Hitachi SU8000, Tokyo, Japan). The electrospun fiber samples were first gold sputter-coated using a Polaron SEM coating system for 1 min at 15 mA. The samples were then loaded into the SEM chamber and imaged using an accelerated voltage of 1.5 kV and a current of 5 µA. Ratios were measured in triplicates. Scanning transmission electron microscopy (STEM, Hitachi SU8000, Tokyo, Japan) was used to verify nanoparticle embedment into the fibers.

### 2.4. Measurement of Magnetic Properties

The magnetic properties of the composite nanofibers were investigated using the quantum design physical property measurement system (PPMS, Quantum Design, San Diego, CA, USA). Inside the measuring chamber, each sample was mounted on rods and connected to the bottom of the helium-cooled dewar on pucks (2.3 cm in diameter). The vibrating sample magnetometry setting was used to measure magnetization versus the magnetic field. Magnetic hysteresis measurements were conducted in the applied magnetic field of ±60 kOe. The magnetic moment per gram of sample versus magnetic field (M-H) loops were recorded from 10 K to 300 K. Magnetic saturation (Ms) was also determined for each sample.

### 2.5. Cell Viability Study

Magnetic nanofiber samples were attached to a 12-mm diameter, circle coverslip using biocompatible silicone-based elastomeric glue (i.e., Kwik-Sil). Each fiber sample was wrapped around the coverslip and glued to the back such that the porous front of the sample was unobstructed and available for cell attachment and infiltration. Samples were sterilized in 24-well plates by incubation in 70% ethanol for 24 h, and then rinsed with DI water and basal medium. Fibroblast 3T3 cells were purchased from the American Tissue Type Culture Collection (Manassas, VA, USA). The growth medium was Dulbecco’s modified Eagle’s medium (DMEM, Life Technologies, Grand Island, NY, USA) supplemented with 10% fetal bovine serum (FBS) and 1% antibiotics (10,000 units/mL of penicillin, and 10,000 µg/mL of streptomycin). 3T3 cells were seeded at a density of 20,000 cells per sample and grown in a humidified incubator (37 °C, 5% CO_2_) for 1, 3, and 5 days. Triplicates of each sample were plated with cells, and cells were seeded on glass coverslips without nanofiber samples to be used as control group. The culture medium was replaced every two days.

The alamarBlue assay (Life Technologies, Grand Island, NY, USA) was used to analyze the cell viability of the 3T3 cells grown on magnetic nanofiber samples. After 1, 3, and 5 days of cell seeding, the coverslips were transferred to new plates, washed twice with phosphate-buffered saline (PBS), and incubated with 10 vol % of alamarBlue reagent in DMEM with 10% FBS for 2 h. Aliquots of 400 µL of assay solution were removed from the wells and transferred to a 96-well culture plate for fluorescent measurements on a Spectra Max Gemini XPS microplate reader (Molecular Devices, Sunnyvale, CA, USA) at *λ*_ex_ 530 nm and *λ*_em_ 590 nm. The relative fluorescent units were converted to a percent of the average values for cells in control wells.

### 2.6. Cell Attachment Study

After the determination of cell viability with the alamarBlue assay, the cells growing on the fiber mats were fixed, and cell attachment was observed by SEM. The cells were washed three times with PBS and fixed with 4% glutaraldehyde (pH 7.4) for 20 min. After fixing, the samples were briefly rinsed with DI water and dehydrated by sequential incubations in 50%, 70% and 100% ethanol at room temperature. The samples were air dried inside a fume hood for 24 h, and then sputter coated with Au for 1 min and 30 s at 15 mA. The samples were imaged with SEM at an accelerating voltage of 10 kV and current of 5 µA.

## 3. Results and Discussion

### 3.1. Mg-Ferrite Nanoparticles and Magnetic Nanofibers

#### 3.1.1. Nanoparticles

**Figure 1:**
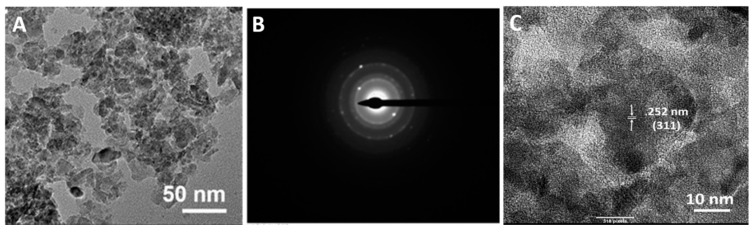
(**A**) Transmission electron microscope (TEM) image of Magnesium ferrite (Mg-ferrite) nanoparticles at lower magnification; (**B**) Corresponding selected area diffraction pattern; and (**C**) TEM image of Mg-ferrite nanoparticles at higher magnification.

Several characterization properties of Mg-ferrite nanoparticles are shown in Figure 1. The morphology of the Mg-ferrite nanoparticles was analyzed using the TEM. The TEM micrograph of the particles is shown in Figure 1A. The digitized micrograph was imported into the program ImageJ (NIH, 1.48v, Rockvile, MD, USA), and the mean particle diameter of the population was determined to be 11.6 ± 3.1 nm. When X-rays interact with atoms of a crystalline, the electron cloud moves, creating waves that result in Bragg diffraction. The wave patterns created by the movement of the electron cloud can be recorded as electron diffraction patterns [31]. The corresponding selected area diffraction pattern for Mg-ferrite nanoparticles is shown in Figure 1B. The electron diffraction patterns for the particles displayed visible rings that corresponded with the (220), (331), (511), (531), (622), (551), and (642) spinel planes, as indicated by the standards listed in the International Center for Diffraction Data (ICDD) Joint Committee for Powder Diffraction Standards data for Mg-ferrite (JCPDS 71-1232). Figure 1C displays a TEM micrograph captured at a higher magnification. With the assistance of Image J, the interplanar space was measured to be 0.252 nm. This d-value was the characteristic of the (311) spinel plane.

**Figure 2:**
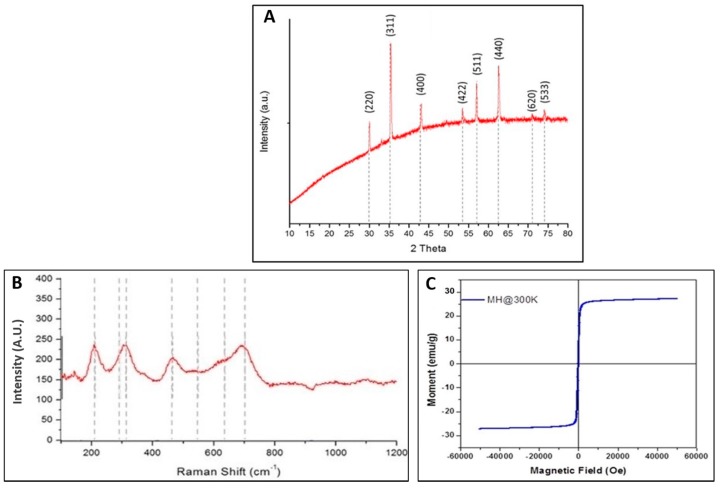
(**A**) X-Ray diffraction (XRD) pattern for the Mg-ferrite nanoparticles; (**B**) Raman spectra for the Mg-ferrite nanoparticles; and (**C**) Magnetization curve of Mg-ferrite nanoparticles.

The crystallinity of the Mg-ferrite powder was further characterized by X-ray diffraction and Raman spectroscopy. Figure 2A depicts the X-ray diffraction pattern for the nanoparticles. The peak position and intensity of the diffraction peaks were compared to Standard Powder Diffraction data (ICDD JCPDS 71-1232). The spectra displayed diffraction peaks at 2-theta (2θ) values of 30.1°, 35.5°, 43.2°, 53.6°, 57.1°, 62.7°, 71.2°, and 74.2°, corresponding to Bragg reflections of (220), (311), (222), (400), (422), (511), (440), (620), and (533) planes of the spinel phase of Mg-ferrite, respectively. No characteristic peak of impurities was observed, and the formation of the face-centered cubic spinel phase of Mg-ferrite was confirmed. The face-centered cubic structure belonged to space group Fd-3m. As a result, the following modes were predicted for the MgFe_2_O_4_ spinel [30]:

$$A_{1g}\left(R\right)+E_{g}\left(R\right)+F_{1g}+3F_{2g}\left(R\right)+2A_{2u}+2E_{u}+4F_{1u}\left(IR\right)+2F_{2u}$$

The five active modes were $A_{1g},\;E_{g}\;\mathrm{and}\;3F_{2g}$. The assignments of the five active modes are listed in Table 1. Aside from the major peaks observed at these modes, smaller peaks have been reported at 212, 291, 407, 483, 550, and 715 cm^−1^ [32].

Raman spectra of the samples were recorded at room temperature within the range of 100–1200 cm^−1^, as shown in Figure 2B. The only features observed in this sample were associated with the spinel structure of Mg-ferrite. Cubic ferrites containing tetrahedral Fe^3+^O_4_ were characterized by a strong Raman band in the 660–720 cm^−1^ region assigned to the A_1g_ mode. Here, the peak at 705 cm^−1^ was assigned to the *A*_1*g*_ mode of the Mg-ferrite nanoparticles [33]. Figure 2C displays the magnetization versus applied field curve for Mg-ferrite sample. The absence of hysteresis and remanence in the hysteresis loop indicated superparamagnetic properties at 300 K for Mg-ferrite nanoparticles [34]. The saturation magnetization for Mg-ferrite nanoparticles was 27.1 emu/g. Holec et al. reported Mg-ferrite nanoparticles by the reverse micelle method with saturation magnetization of 32 emu/g [35]. 

#### 3.1.2. Nanofibers

**Figure 3:**
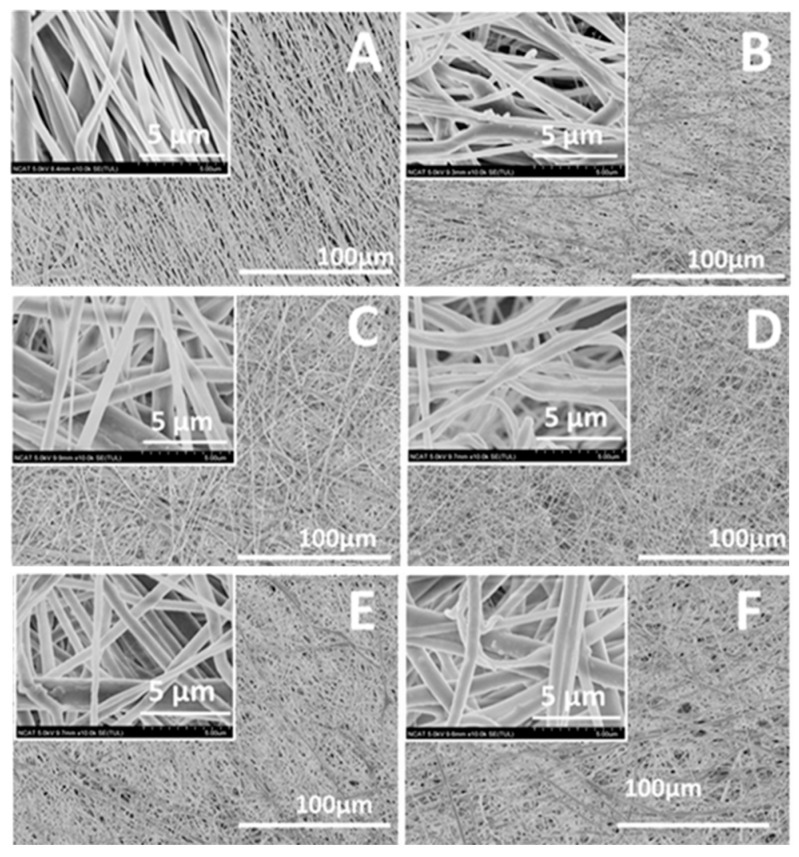
Scanning electron microscopy (SEM) images of magnetic polycaprolactone (PCL)/Aloe vera nanofibers containing (**A**) 0%; (**B**) 5%; (**C**) 10%; (**D**) 15%; (**E**) 20%; and (**F**) 25% Mg-ferrite nanoparticles. Insets show a higher magnification of each corresponding micrograph.

**Figure 4:**
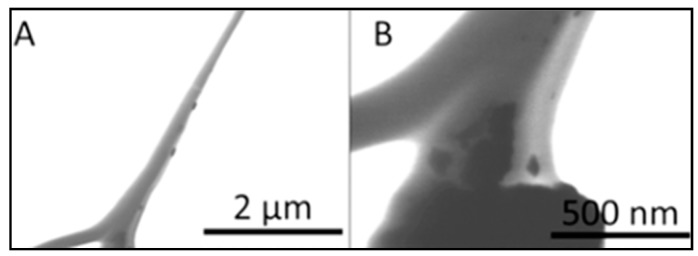
Scanning transmission electron microscopy (STEM) images of magnetic PCL/Aloe vera nanofibers containing 25% Mg-ferrite nanoparticles. Images were captured at (**A**) lower magnification and (**B**) higher magnification.

The fabrication of the Mg-ferrite based nanofiber was achieved by dispersing the Mg-ferrite nanoparticles in a 10 wt % PCL solution prior to electrospinning. To increase the homogeneity of the Mg-ferrite/PCL mixture, 10 wt % Aloe vera solution was added to the PCL/Mg-ferrite mixture. Aloe vera has been reported to have anti-inflammatory effects and enhance cell proliferation [37,38]. The resulting nanofibers are shown in Figure 3. Stable and “bead-free” nanofibers were obtained at the PCL/Aloe vera ratio of 70:30 with 0–25% Mg-ferrite nanoparticles. Mg-ferrite nanoparticles appeared to be embedded in the nanofibers. To further analyze the particle interaction with the nanofibers, STEM was used. Figure 4 displays the STEM image of PCL/Aloe vera fibers with 25% Mg-ferrite. The figure confirmed that the nanoparticles were successfully embedded in the nanofibers.

The successful fabrication of Mg-ferrite based magnetic nanofibers of PCL/Aloe vera was primarily due to the formation of homogenous and viscous solution. Mg-ferrite nanoparticles were blended into the PCL/Aloe solution with suitable viscosity for electrospinning. PCL is a non-ionic polymer that is soluble in a range of solvents, mainly halogenated organic solvents. TFE is a water-miscible fluorinated alcohol. We chose TFE to dissolve PCL, because TFE has been reported to be a good solvent for PCL to create nanofibers by electrospinning [39]. PCL/Aloe vera solutions were prepared by mixing PCL and Aloe vera in TFE and DI water, respectively. Due to the electronegativity of the trifluoromethyl group, TFE exhibits a strong acidic nature. Thus, TFE helped to form heterocyclic complexes between PCL and Aloe vera through hydrogen bonding, which resulted in a homogeneous solution of Mg-ferrite embedded PCL/Aloe suitable for electrospinning [40].

### 3.2. Magnetic Properties of Nanofibers

**Figure 5:**
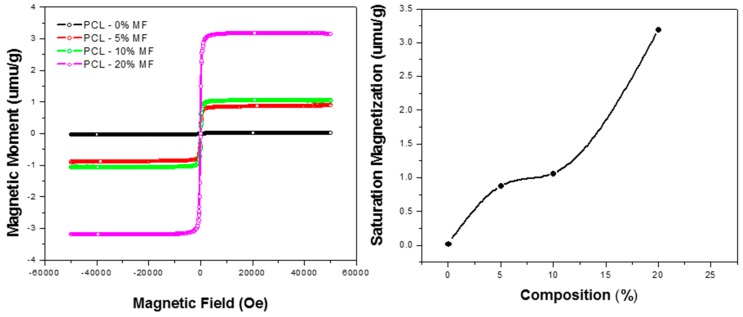
Magnetization curves of Mg-ferrite based PCL/Aloe vera nanofibers.

To confirm the magnetization of the composite nanofibers, magnetization curves of some of the PCL/Aloe vera fibers containing three different percentages of Mg-ferrite particles were analyzed. The magnetization curves are shown in Figure 5. Embedding the Mg-ferrite nanoparticles into the PCL/Aloe vera nanofibers yielded a composite nanofiber that possesses some of the magnetic properties found in the synthesized nanoparticles. All of the composite nanofibers displayed hysteresis and increasing magnetic saturation corresponding to the increasing concentration of Mg-ferrite nanoparticles present in the PCL/Aloe vera fibers. Such near superparamagnetic is well known to be related with the fast-magnetic relaxation of the nanoparticles, as seen in iron oxide-based polymer nanofibers [41]. The fast relaxations were caused by the removal of the polarization of the dipole moments of the ferrite nanoparticles, due to the thermal fluctuation. Since nanoparticles were embedded in the solid nanofibers, the thermal fluctuation did not achieve its full effect. The increasing concentration of the Mg-ferrite nanoparticles in the fiber might have increased the aggregates of nanoparticles. That might increase the magnetic domain size in some spots, and fibers showed some ferromagnetic behavior. The saturation magnetization was 0.024, 0.88, 1.06 and 3.19 emu/g, respectively. The relatively smaller value of saturation magnetization compared with bulk iron is known to be general for magnetic nanomaterials. The confinement of magnetic nanoparticles in a solid polymer matrix might also be responsible [42].

### 3.3. Cell Viability of Magnetic Nanofibers

Cell viability was assessed using an alamarBlue assay. The dye, alamarBlue, is the chemical resazurin. After this blue, non-fluorescent dye enters living cells, mitochondrial reductases reduce resazurin to resorufin, which is pink and fluorescent [43]. The amount of dye is proportional to cellular metabolic activity, which itself is proportional to cell number. Thus, comparison to control samples provides a relative measure of cell number. A blend of PCL/Aloe vera in 70:30 ratio was investigated in this study. In a separate study, we found that stable and completely “bead-free” solid nanofibers were obtained by electrospinning at a PCL/Aloe vera ratio of 70:30 or higher [5]. We also found that PCL/Aloe vera nanofiber samples with a ratio of 70:30 showed greater cell viability compared with samples with ratios of 80:20, 90:10 and 100:0.

**Figure 6:**
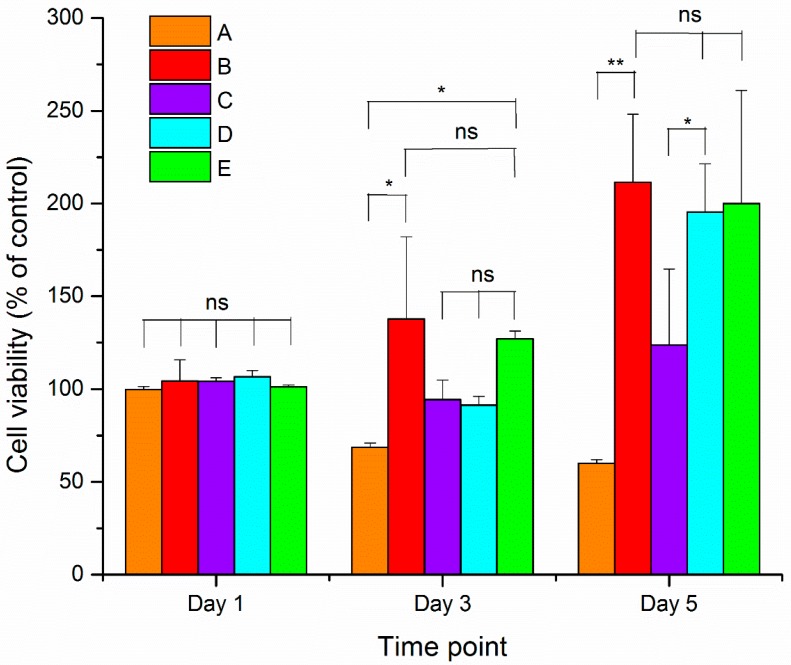
Cell viability results of 3T3 cells grown on Mg-ferrite based PCL/Aloe vera fibers. A and B represent the 100:0 and 70:30 PCL/Aloe vera fibers with 0% Mg-ferrite nanoparticles, respectively. C, D and E represent 70:30 PCL/Aloe vera fibers containing 5%, 15% and 25% Mg-ferrite nanoparticles, respectively. Cell viability data were analyzed for statistical significance using paired *t*-test (*n* = 3). A statistical significant of *p* < 0.05 is indicated by * and *p* < 0.005 by **. Statistical insignificant of *p* > 0.05 is indicated by ns.

Figure 6 shows the cell viability of 3T3 cells on nanofibers after 1, 3 and 5 days of cell seeding. After one day of cell seeding, there were no significant changes in the relative levels of alamarBlue between 3T3 cells grown on the nanofibers and on the control substrate (i.e., glass coverslip). Thus, cell numbers and activity on the nanofibers were not different after one day. After three days of cell seeding, the PCL/Aloe vera nanofiber samples containing 0 and 25% Mg-ferrite nanoparticles displayed significant cell proliferation, but there were no significant changes in the samples containing 5% and 15% Mg-ferrite nanoparticles. After five days of cell seeding, the PCL/Aloe vera nanofiber samples containing 0%, 15% and 25% magnesium ferrite nanoparticles displayed significant cell proliferation. The PCL/Aloe vera nanofiber samples with a ratio of 100:0 displayed the least cell viability at both time points of three and five days of cell seeding. 

Thus, the combination of synthetic PCL with natural polymer Aloe vera at a 70:30 ratio significantly improved the cellular compatibility of 3T3 cells on the nanofibers, which substantiates that the enhancement of hydrophilicity of Aloe vera can provide adequate support for 3T3 cell growth and proliferation. The PCL/Aloe vera nanofiber samples with a ratio of 70:30 containing 25% magnesium ferrite nanoparticles displayed similar characteristics. A considerable number of cells proliferated and remained viable on the PCL/Aloe vera fibers containing 25% Mg-ferrite nanoparticles. All the PCL/Aloe vera samples with a ratio of 70:30 containing 5%, 15% and 25% Mg-ferrite nanoparticles were non-toxic relative to control substrate.

### 3.4. Cell Attachment of Magnetic Nanofibers

**Figure 7:**
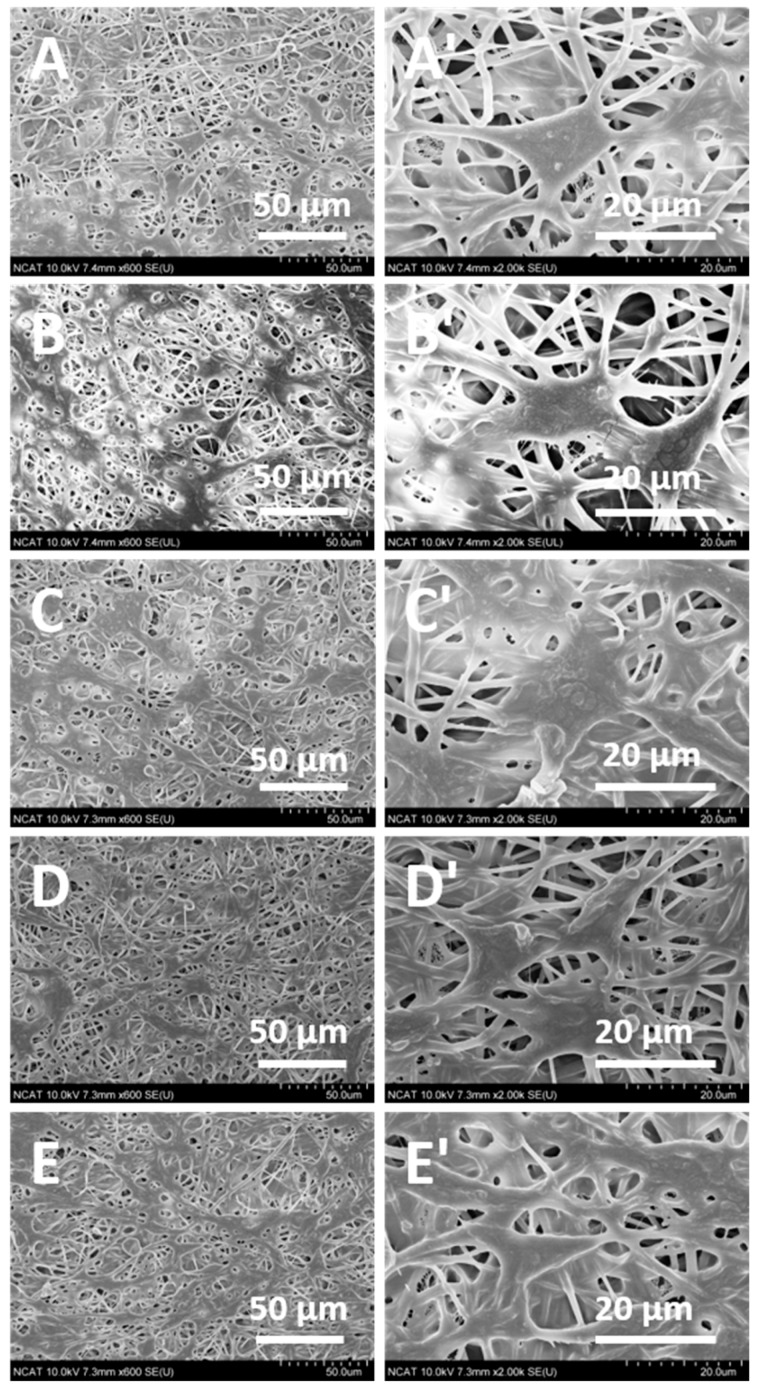
SEM images displaying the morphology of 3T3 fibroblast cells seeded on Mg-ferrite based PCL/Aloe vera nanofibers after three days of cell seeding. (**A**,**B**) represent 100:0 and 70:30 PCL/Aloe vera nanofibers with 0% Mg-ferrite nanoparticles, respectively. (**C**–**E**) represent 70:30 PCL/Aloe vera fibers containing 5, 15, and 25% Mg-ferrite nanoparticles, respectively. Images (**A’**–**E’**) are higher magnification images of (**A**–**E**), respectively.

To further evaluate the cellular compatibility of the Mg-ferrite based PCL/Aloe vera nanofibers, cell adhesion and spreading, as well as cell interactions with the nanofibers, were investigated by SEM. Figure 7A,B showed the SEM images of fibroblast 3T3 cells grown on the PCL/Aloe vera nanofibers with ratios of 100:0 and 70:30 after three days of cell seeding. The cells attached well and formed cell clusters on the nanofibrous structure. The SEM micrographs showed that the fiber architecture guided the development of fibroblast growth. The cells showed greater spreading on the PCL/Aloe nanofiber samples with ratios of 70:30 compared to the PCL/Aloe nanofibers with ratios of 100:0. The PCL/Aloe vera nanofiber samples showed attachment of cells to the surfaces by numerous, long filopodia. The filopodia of the cells tended to attach to, and grow along, the polymer nanofibers, whose diameter is similar to that of the filopodia. Such cellular morphology is indicative of a favorable interaction of fibroblasts with the nanofibers. 

In terms of PCL/Aloe vera nanofibers containing 5%, 15% and 25% Mg-ferrite nanoparticles (Figure 7C–E), the cell–fiber interaction cannot be distinguished quantitatively using SEM micrographs. On these samples, a large number of cells divided and formed a continuous cell layer that covered the nanofiber samples. On the PCL/Aloe nanofiber samples with a ratio of 70:30 containing 0% and 25% Mg-ferrite nanoparticles, the cells exhibited better alignment and presented as flat spindle shapes on the fibers, which indicates greater interaction and boding between the cells and fibers.

Magnetic nanoparticles have been widely used for bioimaging, drug delivery and hyperthermia treatment. However, recent developments of magnetic nanoparticles demonstrate promise towards tissue regeneration and growth [44]. To prepare the most effective magnetic nanoparticles for tissue engineering applications, particle characteristics including size, surface chemistry, magnetic properties, and cellular compatibility need to be fully investigated. Mg-ferrite is a well-known spinel ferrite reported for intensive research to utilize its properties for tissue engineering. Three common methods for preparing Mg-ferrite nanoparticles are gel combustion, co-precipitation, and reverse micelle. In this study, Mg-ferrite nanoparticles were prepared using the reverse micelle method. Reverse micelle is a relatively better method, because nanoparticles obtained from gel combustion and co-precipitation are highly agglomerated [30]. Nanofibrous scaffolds for neural tissue engineering are designed to support the three-dimensional growth of neuronal cells and regenerated nerve fibers. Neurotrophic factors (e.g., magnesium) are added to neuronal cultures in order to enhance nerve fiber regeneration, neuronal cell growth, and maturation [45]. Recently, a few attempts have been made to prepare magnetic nanoparticles-based electrospun nanofibers [20,41,42,46]. Although these recent efforts in fabricating magnetic nanofibers are encouraging, much remains to be explored and improved, particularly in regards to neural tissue engineering applications. In this study, the morphology, structural and magnetic properties, and cellular compatibility of Mg-ferrite based PCL/Aloe vera nanofibers were explored to substantiate the potential of these fibers for biomedical applications.

## 4. Conclusions

Mg-ferrite based PCL/Aloe vera nanofibers were successfully fabricated by an electrospinning technique. Mg-ferrite nanoparticles were first synthesized via the reverse micelle method and properties such as their morphology, crystallinity, and magnetic properties were characterized. The reverse micelle method was highly reproducible and yielded uniform magnetic nanoparticles. The nanoparticles were then dispersed in the PCL/Aloe vera solution. The blend solution of Mg-ferrite/PCL/Aloe vera was electrospun to produce magnetic nanofibers. The resulting nanofibers exhibited good morphological uniformity, structural integrity, magnetic strength, and cellular compatibility. Mg-ferrite based PCL/Aloe vera nanofibers are not expensive to produce, and are easy to synthesize and scale up for various biomedical applications. In addition to optimizing processing and characterization techniques, in vivo cytocompatibility screening remains to be explored. 

## Figures and Tables

**Table 1:** Raman modes of MgFe_2_O_4_ [36].

Raman Modes (cm^−1^)	Assignment
217	$F_{2g}$
333	$E_{g}$
486	$F_{2g}$
554	$F_{2g}$
715	$A_{1g}$
